# The Science and Philosophy of the Brain and the Future of Neuroscience

**DOI:** 10.3390/biology13080607

**Published:** 2024-08-11

**Authors:** Julian Paul Keenan

**Affiliations:** Cognitive Neuroimaging Laboratory, Department of Biology, Montclair State University, 320 Science Hall, Montclair, NJ 07043, USA; keenanj@montclair.edu

The future of neuroscience is epitomized in this Special Issue of *Biology*, titled “Representations and Distributions of Higher Brain Functions at a Functional, Anatomical, and Neuronal Level”. This issue is forward-thinking, and it comprises the work that will be the basis of the next decade of neuroscience. These articles are from the most prominent scholars from across the globe and address a wide range of topics, from PTSD to neurofeedback to free will, depression, artificial networks, and brainprint recognition. 

Neuroscience has always been diverse, effective, and complex, and we see that here in these articles. In this issue, we find a mix of applied technology and deep philosophy acting together to address the practical. Sepahvand and colleagues discuss the basolateral amygdala [1] and its role in threat-based learning. The article begins with a wonderful summary of many decades of neuroscience research but then takes us into the future with an amazing array of new findings, including DNA methylation and protein synthesis as potential biomarkers. This work compliments that of Skolarki and fellow researchers [2], who examine PTSD at a mostly genetic and protein level, modeling the interconnectivity of numerous factors. A short but critical mathematical modeling section is provided, and this work will serve as a template for much to follow. These two articles serve as wonderful guides to the past, present, and future of stress, fear, and PTSD work.

Lawson and her group ask if it can be called ‘free will’ [3] if it can be so easily manipulated. They find that people seem to be unaware of their motives and ‘make-up’ causes. They suggest that free will may be more fragile than once thought, again showing that neuroscience is not afraid of big questions. Nevertheless, neuroscience is practical. Nguyen and the group of researchers, mainly focused in Minnesota, used neurofeedback in the treatment of depression [4]. This group, with Quevedo as its mentor, has employed neuroscience to address many relevant issues, including teen suicide in depression. In this paper, the focus is neurofeedback and its effectiveness.

Artificial neural networks [5] and brainprint [6] are the focus of the final papers. Here we see a focus on self-recognition, a topic of interest to philosophers and AI researchers. A wonderful summary is written, followed by the assertion that entropy may be critical. Future research will follow up on these thoughts. Future research will also address the limitations of artificial neural networks (ANN). Specifically, applying the ANN to organic brain tissue is not simple or easy, but also not impossible. This review is a great guide to what we know and what still stands in the way.

I may live to see us decipher the neural correlates of non-human language or the employment of optogenetics to treat all forms of brain disease. With the work that is within this issue, we will all live to see amazing breakthroughs in neuroscience.
